# Autonomic adaptations mediate the effect of hydration on brain functioning and mood: Evidence from two randomized controlled trials

**DOI:** 10.1038/s41598-019-52775-5

**Published:** 2019-11-11

**Authors:** Hayley A. Young, Alecia Cousins, Stephen Johnston, John M. Fletcher, David Benton

**Affiliations:** 1 Department of Psychology, Swansea University, Swansea, SA2 8PP Wales UK; 2 PepsiCo, 100 Summit Lake Drive, Valhalla, NY USA

**Keywords:** Striatum, Hypothalamus, Human behaviour

## Abstract

Dehydration (water loss >2.0% of body weight) has significant negative effects on physical and mental performance. In two studies the effects of minor hypo-hydration (water loss <1.0% of body weight) on CNS function, mood and cardiovascular functioning were measured. Study 1: On two mornings twelve male participants were exposed to a temperature of 30 °C for four hours and either did or did not drink two 150 ml glasses of water during that time. Study 2: Fifty-six (25 M) individuals were exposed to the same 30 °C environment and randomly allocated to either drink (2 × 150 ml) or not drink. When not given water 0.59% (Study 1) and 0.55% (Study 2) bodyweight was lost. Participant’s heart rate variability (HRV) was measured, and they rated their thirst and mood. In study 1, participants participated in an fMRI protocol during which they completed a modified version of the Paced Auditory Serial Addition Test (PASAT), at the end of which they rated its difficulty. Decreases in fMRI BOLD activity in the orbito-frontal cortex, ventral cingulate gyrus, dorsal cingulate cortex, hypothalamus, amygdala, right striatum, post-central gyrus and superior parietal cortex were observed when participants were hypo-hydrated. These deactivations were associated with reduced HRV, greater perceived effort, and more anxiety. In study 2 declines in HRV were found to mediate the effect of hypo-hydration on ratings of anxiety. These data are discussed in relation to a model that describes how autonomic regulatory and interoceptive processes may contribute to the affective consequences of minor hypo-hydration.

## Introduction

It has been assumed that small variations in hydration status, in what might be described as the normal day to day range (water loss <1.0% of body weight), have no significant influence on higher mental functions such as cognition and mood^1^. Indeed, where dose – response relationships have been assessed, the largest psychological effects appear to occur with moderate levels of dehydration (water loss 2.0–5.0% of body mass), although findings may depend on the method used to induce dehydration^2^. In healthy individuals such large changes in hydration status are rare and are associated with water deprivation and prolonged exposure to hot environmental temperatures and/or exercise. Therefore, the relevance of such findings to the majority of the population is unclear. It is argued that thirst and homoeostatic mechanisms keep the hydration status of those with access to water and living in temperate environments within a narrow range. However, it is plausible that even when fluid balance is successfully maintained, certain counter-regulatory mechanisms may themselves have psychological consequences – this possibility is yet to be explored. For the first time the present studies report that with as little as a 0.6% decrease in body mass, changes in cardiovascular and autonomic functioning (heart rate variability) negatively influence mood. These data highlight novel mechanisms that may contribute to the affective consequences of minor hypo-hydration.

The adverse consequences of dehydration (water loss >2.0% of body mass) have been well described and include declines in physical and mental performance, and increased blood pressure and heart rate^3^. Questioning recieved wisdom we recently reported that participants exposed to a temperature of 30 °C for four hours lost less than 1% of their body mass but experienced a significant decline in memory and focused attention; effects that were reduced by drinking water^4,5^. In an experimental protocol that tested performance on a simulated driving task, hypo-hydration (loss of 1.1% body mass) caused increased errors and changes in EEG alpha and theta waves indicative of drowsiness^6^. Interestingly, the most commonly observed consequences of minor hypo-hydration may be subjective^2,7,8^. For example, in the study by Benton, *et al*.^4^, an increase in thirst was associated with a decline in subjective energy and increased anxiety and depression. Therefore, given that fluid balance is highly regulated^9^, there is a need to understand the mechanisms underpinning these psychological consequences of minor hypo-hydration.

The physiological regulation of fluid balance is relatively well understood^10^, several forebrain and brainstem circuitries interact with peripheral neural and humoral signals to collaboratively maintain the osmolality and volume of fluids. One counter-regulatory mechanism involves changes in cardiovascular and autonomic functioning: hypo-hydration induces cardiovascular strain^3^, for every 1% decrease in body mass during exercise there is an increase in heart rate of 3.29 beats per min (bpm)^11^. Dehydration decreases the volume of blood (absolute hypovolemia), reducing cardiac output, and necessitating an increase in heart rate to maintain blood pressure. When there is a simultaneous elevation in skin temperature blood vessels are dilated resulting in relative hypovolemia; an inadequate distribution of blood volume between the periphery and core. These counter-regulatory changes, while part of normal body fluid homeostasis, may alter psychological functioning.

For example, fluid consumption has been shown to facilitate peripheral and cerebral perfusion when exposed to physiological^12^, orthostatic^13^ or psychological^14^ stress. Even in the absence of hypo-hydration, water ingestion is followed by an increase in cardiac vagal control^15^; an effect thought to counteract the pressor effects of sympathetic activation. Interestingly, neuroimaging studies that have attempted to understand our experience of hypo-hydration and thirst find changes in regions of the brain often associated with autonomic and cardiac control^16,17^; that is regions comprising the central autonomic network^18^. For example, Farrell, *et al*.^19^ observed that a state of osmotic thirst (induced with hypertonic infusions) was related to changes in blood flow to the somatosensory and motor cortices, prefrontal cortex, anterior mid cingulate cortex, and superior temporal gyrus.

According to the neuro-visceral integration model of emotion regulation^18^, the central autonomic network mediating sympathetic and parasympathetic control include regions of the limbic forebrain such as the subgenual and pregenual cingulate, amygdala/ventral striatum and medial prefrontal cortex^20^. Essentially the prefrontal cortex exerts inhibitory GABAergic control over the limbic regions of the brain that ultimately control heart rate^20,21^. This implies that in the presence of a physiological or psychological stressor that necessitates an increase in heart rate, there will be an associated decline in prefrontal neural activity. The relevance is that individual differences in vagal tone (measured using heart rate variability (HRV)) predict mood and cognitive performance, especially in tasks associated with the prefrontal and cingulate cortices^22–25^. Thus, it is plausible that cardiovascular and autonomic adaptations, may explain the affective consequences of minor hypo-hydration.

Although neuroimaging studies have attempted to understand our experience of thirst while at rest, only one study has considered the effect of hydration on brain functioning during a cognitive task. Kempton *et al*.^26^ studied the effects of fluid restriction during a thermal exercise protocol on fMRI BOLD responses to the Tower of London task. Participants lost 1.64% bodyweight and heart rate was about 20 bpm higher during dehydration. There were no effects on two subjective rating of sedation although this may be explained by the confounding effects of physical activity. Similarly, performance on the task was not altered, participants had a greater BOLD response in the fronto-parietal cortex when dehydrated; an effect interpreted as reflecting a need for a higher level of neuronal activity to achieve the same performance level. Kempton *et al*.^26^ fMRI analysis used a region-of interest approach which specifically examined task-related regions of activation. Therefore, it remains undetermined whether there were effects on brain regions linked with autonomic and cardiac control. In addition, although differences in the degree of bodyweight lost and core temperature were correlated with differences in neural activity, heart rate was not. Therefore, the possibility that alterations in cardiovascular or autonomic functioning mediate the association between hydration and psychological/affective functioning remains unexplored.

The aim of the present two studies was to determine whether cardiovascular and autonomic adaptations, may explain the affective consequences of minor hypo-hydration. Study one reports, for the first time, that change in blood flow to particular neural systems, as indicated by fMRI, can be observed with as little as a 0.6% decrease in body mass. It was shown that hydration deficits resulted in changed activity in the blood flow to the ventral cingulate gyrus (vCG) and medial orbito-frontal cortex (mOFC) that reflected autonomic adaptations to the physiological challenge of hypo-hydration (changes in HRV). In addition, it was observed that these adaptations correlated with changes in affect. Study two extends these novel findings showing that mild hypo-hydration reduced HRV that in turn mediated effects on mood.

## Statistical Analysis

### Hydration indices (Study 1 and Study 2)

**Figure 1:**
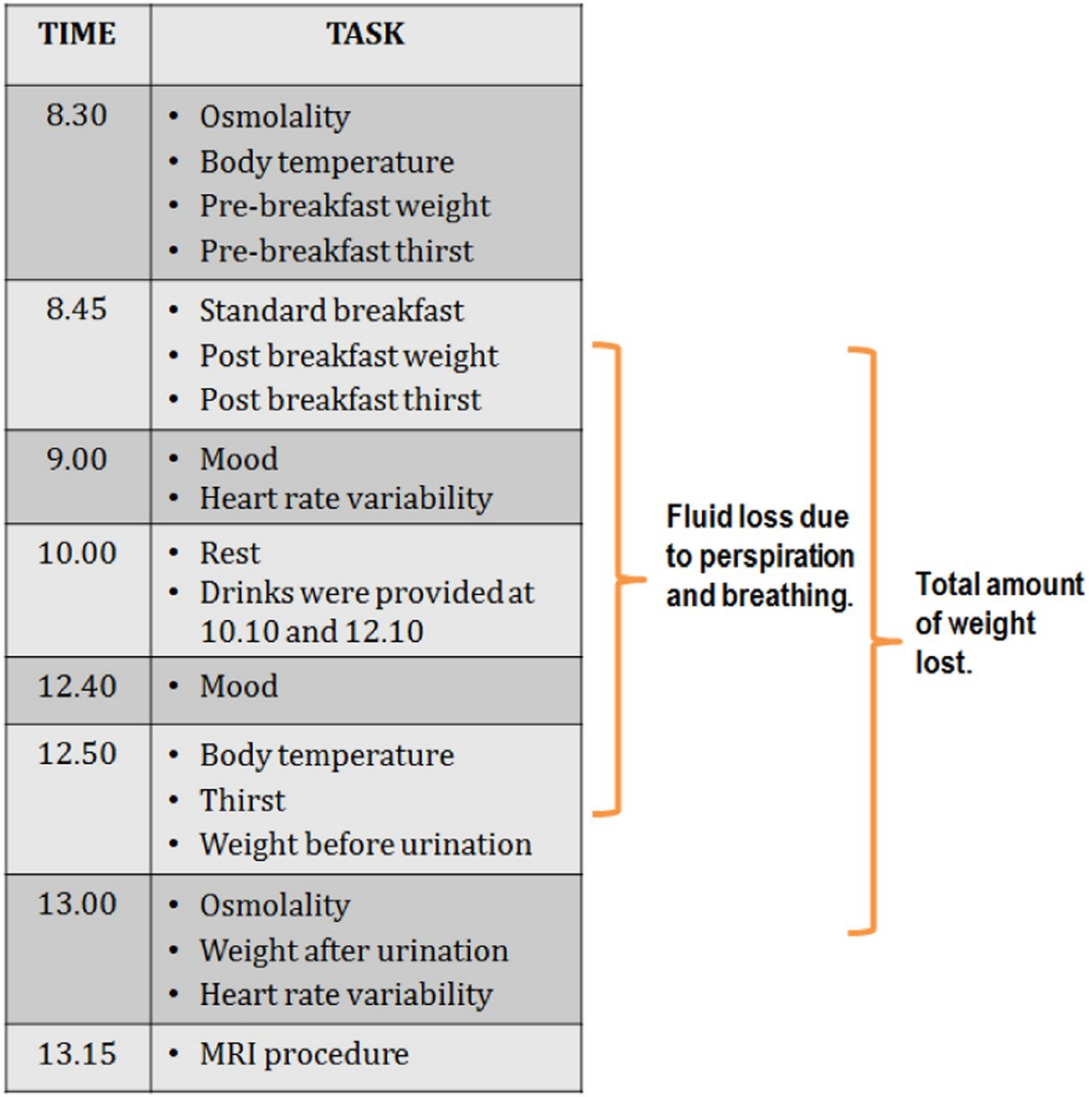
The experimental procedure.

Fluid loss due to perspiration and breathing (hereafter referred to as perspiration rate) was estimated as the percentage change in body mass from baseline to the end of the session prior to urination (Fig. 1). Total body mass lost, including urination, was calculated as total percentage change in body weight. For body temperature and osmolality, change scores were calculated (end of the morning minus baseline). Similarly, thirst change scores were calculated (end of morning minus thirst following the standard breakfast).

### Heart rate variability (Study 1 and Study 2)

Interbeat (RR) intervals were recorded at rest for five minutes at the start of the procedure and again at the end. Interbeat interval data were analysed using Kubios HRV Analysis Software 2.0^27^ (The Biomedical Signal and Medical Imaging Analysis Group, Department of Applied Physics, University of Kuopio, Finland). Data were visually inspected for artefacts caused by ectopic beats, poor conductivity etc. A very low correction threshold was chosen for artefact correction (0.45 from local average) so not to distort natural variability. Less than 1% of beats were identified as artefacts. Time domain HRV indices included mean R-R interval (a measure of basic heart rate), the standard deviation of normal to normal R-R interval (SDNN) (measures total variability in the series) and the root mean square of the standard deviation (RMSSD) (a measure of parasympathetic nervous system activity). To determine the effect of hydration, change scores were calculated (end of the morning minus baseline) for each index.

### Subjective ratings (Study 1 and Study 2)

For ratings of anxiety, depression and energy change scores were calculated (end of morning minus mood following the standard breakfast).

### fMRI analysis (Study 1)

BOLD sensitive echo planar images were collected as participants performed the arithmetic task (TE = 30 ms, TR = 3000 ms, flip angle = 90, axial plane, interleaved acquisition, voxels = 3 mm 3 mm 3 mm, slices = 44). In each of the three separate runs a total of 135 volumes were collected. Additionally a high-resolution magnetization-prepared rapid acquisition with gradient echo (MPRAGE) 3D volume image was acquired for each participant (TE = 4.82 ms, TR = 2500 ms, flip angle = 7, sagittal plane, 1 mm3 isotropic voxels, 179 slices).

Analyses were conducted using BrainVoyager QX 2.8^28^ (Brain Innovations B.V., the Netherlands). Data were pre-processed as follows: 3D motion correction (6 degrees of freedom) with sinc interpolation to account for participant head movement during the scanning procedure, linear trend removal and temporal high pass filtering (low cutoff: 3 cycles per run), and spatial smoothing using a 6 mm Gaussian FWHM filter. Data were normalised to the standard Talairach space^29^ using sinc interpolation prior to group analysis. The group analysis involved estimating task-correlated activity using a general linear model (GLM) approach. The predictors for the GLM were created by convolving the timecourse of the stimulation periods (arithmetic operation) with a canonical HRF response. Each participants beta estimates, obtained via the GLM, were then input into a voxel wise second level random effects analysis. The threshold set for statistical significant was a voxel-wise p < 0.05, with an additional multiple comparison correction applied via cluster level thresholding using the Monte Carlo simulation tool implemented in BrainVoyager QX 2.8 (also with a statistical acceptance threshold of p < 0.05).

### Arterial spin labelling (ASL) analysis (Study 1)

For each participant an arterial spin labelling (ASL) sequence was run after the functional imaging experiment had been completed. The ASL sequence parameters were TR = 2500 ms, TE = 12 ms, in-plane resolution = 4 mm × 4 mm, 90 averages for each label and control pair, slice thickness = 8 mm with a 2 mm gap, 9 slices were collected. The labelling time was 700 ms and the post-labelling time was 1800 ms. The labelling plane was placed 18.8 mm inferior to the bottom slice. Scans were planned such that the ASL were in an AC-PC orientation, with the topmost scan aligned with the most superior aspect of the participant’s brain. ASL data were 3D motion corrected and normalised to Talairach space. Relative perfusion values were obtained for each participant and condition using the ASL toolbox implemented in BrainVoyager QX 2.8. Significant differences between dehydrated and water conditions were assessed using paired t-test of the relative perfusion values.

## Results: Study 1

### Verification of the hydration paradigm

The effect of drinking on percentage total weight loss, perspiration rate and changes in osmolality, thirst and body temperature were analysed using RMANOVA. As expected when participants did not drink water they lost more weight both before (F(1,11) = 214.853, p < 0.0001, η_p_^2^ = 0.951) and after (F(1,11) = 34.777, p < 0.0001, η_p_^2^ = 0.760) urination; in total an average of 0.6% of their body mass (Table 1). Hypo-hydration resulted in a significant increase in urine osmolality (F(1,11) = 23.89, p < 0.001, η_p_^2^ = 0.749) and participants tended to be more thirsty when they did not drink (F(1,11) = 4.178, p < 0.07, η_p_^2^ = 0.317). There was no difference in participants body temperature depending on whether or not they consumed water (F(1,11) = 1.887, p = 0.197, η_p_^2^ = 0.146).

**Table 1:** The effect of drinking compared with not drinking on hydration indices and heart rate variability.

	Study 1		Study 2	
	No Water	Water	No Water	Water
Weight lost before urination (%)	**−0.40** (**0.01**)**	**−0.01** (**0.03**)**	**−0.32** (**0.04**)**	**−0.08** (0.04**
Weight lost after urination (%)	**−0.59** (**0.03**)**	**−0.26** (**0.05**)**	**−0.54** (**0.05**)*	**−0.36** (**0.05**)*
∆ Osmolality (mOsm/kg)	**219.77** (**57.50**)**	**−190.00** (**55.47**)**	**116.88** (**33.66**)*	**−9.03** (**33.66**)*
∆ Temperature (°C)	0.75 (0.11)	0.50 (0.19)	0.32 (0.07)	0.32 (0.07)
∆ Thirst (VAS)	24.40 (4.64)	12.30 (5.92)	18.82 (6.62)	5.96 (6.62)
∆ R-R interval	**17.75** (**19.47**)*	**88.42** (**31.90**)*	**20.90** (**27.44**)**	**160.94** (**27.44**)**
∆ SD of R-R interval	**12.59** (**10.43**)*	**−12.80** (**5.68**)*	**−5.34** (**4.38**)*	**8.77** (**4.38**)*
∆ RMSSD	**3.87** (**6.96**)*	**13.38** (**5.89**)*	**−0.67** (**4.44**)*	**13.21** (**4.44**)*

Data are mean (s.e.) for change scores across each morning (end of the morning minus baseline) for study 1 and study 2. In both studies, compared to when they drunk, when participants did not drink they lost more weight both before and after urination and their osmolality increased. They had a significantly greater increase in HRV (RR interval, SDNN, RMSDD) when they drunk compared to when they did not drink. ∆ Change across the morning, *Water vs. No water significant at p < 0.05, **Water vs. No water significant at p < 0.001. RR, interbeat interval, SDNN, standard deviation of normal to normal R-R interval, RMSSD, root mean square of the standard deviation.

### Heart rate variability

**Figure 2:**
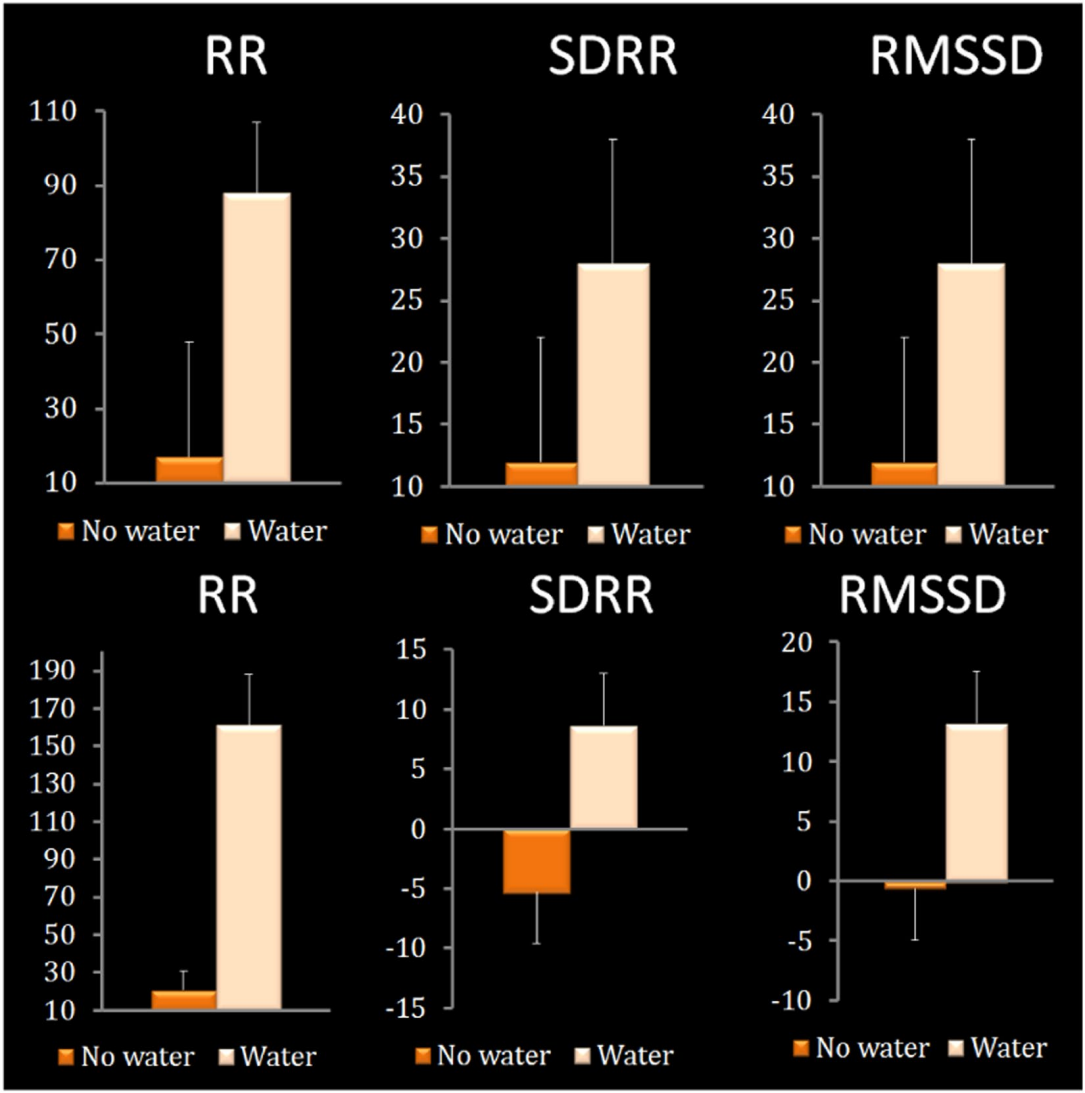
The effect of drinking water on the average length of the RR interval, SDNN and RMSSD. Data are the changes (end of the morning minus baseline) for each index compared across the two conditions. Top panel: Study 1. When participants were hypo-hydrated they had a lower R-R (i.e a higher HR) (p < 0.03), lower SDRR (i.e a lower HRV) (p < 0.01) and reduced parasympathetic activity (i.e. lower RMSSD) (p < 0.03). Bottom panel: Study 2. If water was consumed participants had an increase in their average R-R interval (p < 0.001), a larger standard SDNN (p < 0.027), and a larger RMSSD (p < 0.031). RR, interbeat interval, SDNN, standard deviation of normal to normal R-R interval, RMSSD, root mean square of the standard deviation.

The effect of drinking water on heart rate variability was considered using RMANOVA where change scores (end of the morning minus baseline) from each day were entered (Table 1; Fig. 2). When participants consumed water they had a larger increase in their average R-R interval, that is they had a lower heart rate (F(1,11) = 5.766, p < 0.03, η_p_^2^ = 0.344). In addition, when participants had drunk water they had higher heart rate variability as shown by a larger standard deviation of the R-R interval (F(1,11) = 9.120, p < 0.01, η_p_^2^ = 0.395), and a larger root mean squared of standard deviation of R-R interval (a measure of vagal activity) (F(1,11) = 6.128, p < 0.03, η_p_^2^ = 0.357).

### Cognitive performance in the scanner

Data were analysed using RMANOVA with condition (Water, No water) as a repeated measures factor. Main effects are shown in Table 2. The number of non-responses (F(1,11) = 0.277, p = 0.609, η_p_^2^ = 0.020), reaction times (F(1,11) = 1.884, p = 0.197, η_p_^2^ = 0.146) and accuracy (F(1,11) = 0.549, p = 0.474, η_p_^2^ = 0.048) did not depend on whether participants had drunk (Table 2).

**Table 2:** Effects of drinking compared with not drinking on performance, perceived difficulty and mood (inside and outside the scanner) in study 1 and study 2.

	Study 1		Study 2	
	No Water	Water	No Water	Water
N^o^ of non-responses	20.08 (7.50)	16.91 (3.21)	—	—
Reaction time (RT in s)	0.611 (0.02)	0.627 (0.01)	—	—
Accuracy (N^o^ correct)	200.08 (9.36)	206.83 (3.93)	—	—
Perceived difficulty (VAS)	181.16 (12.0)	175.00 (12.0)	—	—
Anxiety (VAS)	103.50 (14.6)	90.20 (17.78)	—	—
Happiness (VAS)	148.60 (7.77)	157.50 (7.52)	—	—
Energy levels (VAS)	112.30 (9.69)	132.80 (13.72)	—	—
∆ Composed (VAS) (outside the scanner)	−9.50 (2.95)*	−0.75 (3.26)*	−16.39 (5.32)*	−0.07 (5.32)*
∆ Energetic (VAS) (outside the scanner)	−14.33 (5.94)	−2.33 (5.20)	−16.03 (4.59)	−16.50 (4.59)
∆ Depressed (VAS) (outside the scanner)	−7.91 (3.97)	0.41 (4.23)	−12.12 (3.56)	−7.85 (3.56)

Data are mean (s.e.). VAS, Visual Analogue Scale. *Water vs. No water significant at p < 0.05.

### Subjective ratings

The subjective responses taken inside and outside the scanner were analysed using RMANOVA with condition (Water, No water) as a repeated measures factor. There were no effects of drinking on ratings of anxiety (F(1,11) = 0.693, p = 0.427, η_p_^2^ = 0.025), happiness (F(1,11) = 1.875, p = 0.204, η_p_^2^ = 0.172), energy (F(1,11) = 2.047, p = 0.186, η_p_^2^ = 0.185) or difficulty (F(1,11) = 0.123, p = 0.733, η_p_^2^ = 0.011) (Table 2) while participants were in the scanner. However, before entering the scanner (whilst still in the 30 degree environment) participants were significantly less composed when they did not drink water; an effect prevented by drinking (F(1,11) = 4.978, p < 0.047, η_p_^2^ = 0.312). Participants also tended to be less depressed (F(1,11) = 4.211, p < 0.065, η_p_^2^ = 0.277), and more energetic (F(1,11) = 2.906, p < 0.116, η_p_^2^ = 0.209) if they drunk water but these effects did not reach significance.

### Brain functioning

#### fMRI results

**Figure 3:**
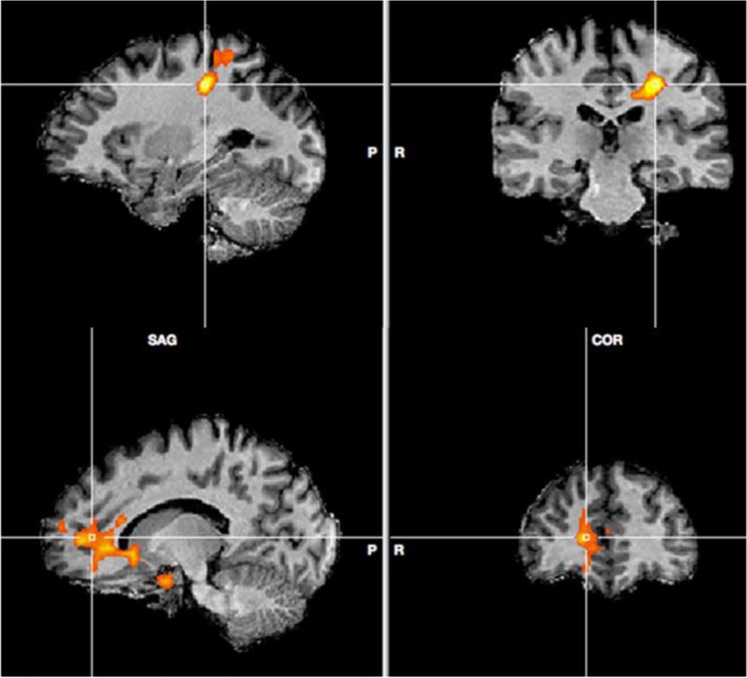
Peak activity in PoCG (top) and mOFC (bottom) water >hypo-hydration.

**Figure 4:**
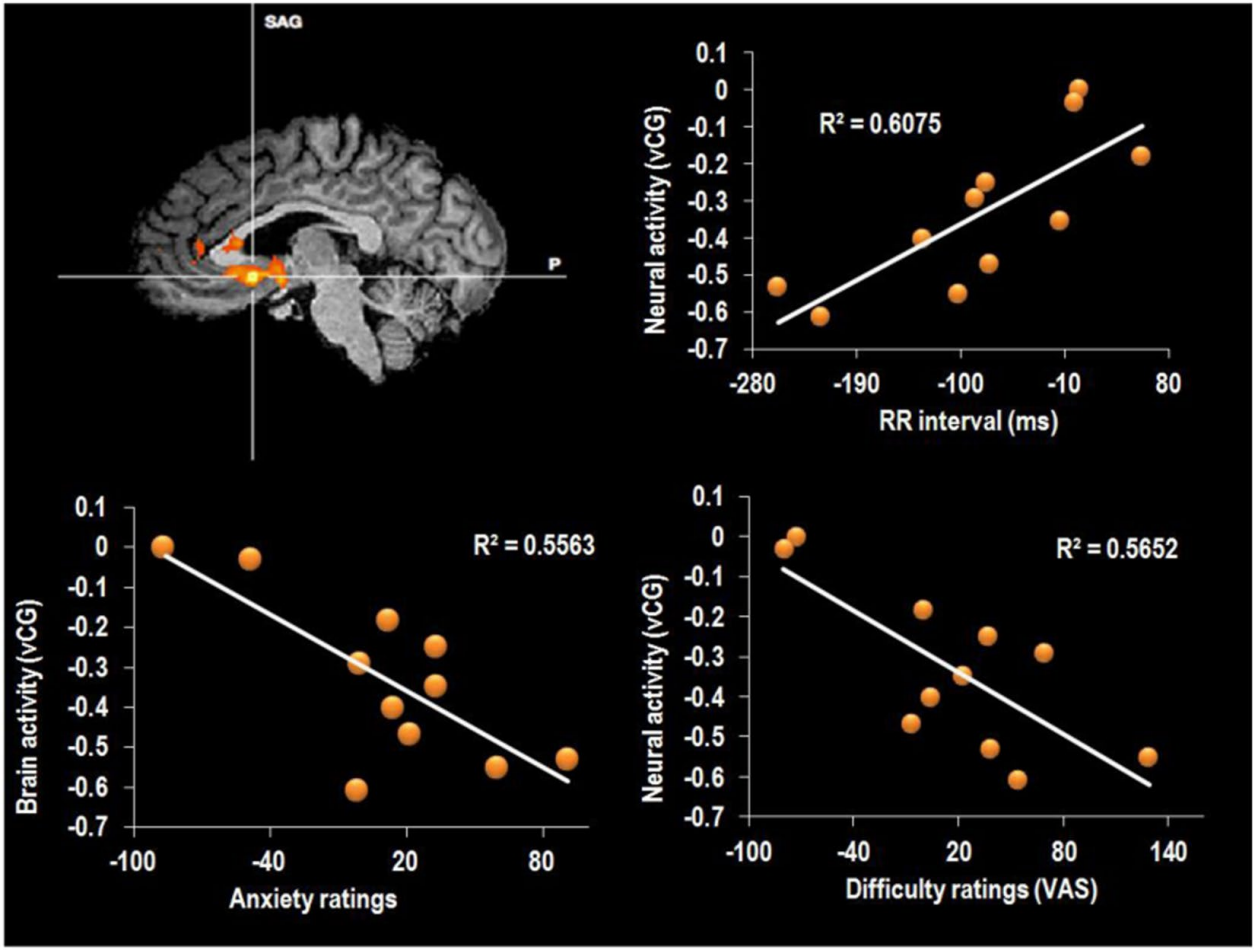
(**A**) Peak activity in vCG water >hypo-hydration. Correlation between neuronal activity in the vCG and (**B**). RR interval (**C**). anxiety and (**D**). difficulty ratings. Data are Pearson’s r coefficient for difference scores (no water – water).

A comparison of activation during the arithmetic task in the water compared to the dehydration condition revealed several clusters that achieved statistical significance. A large cluster was centred on the ventral cingulate gyrus (Talairach co-ordinate: 4, 14, −6; Fig. 4), that extended into both the hypothalamus and striatal brain regions. Specifically, striatal activity was observed in the bilateral caudate nucleus and putamen. The same cluster also spread inferiorly to right amygdala. To the anterior aspect the activity spread into the medial orbito-frontal cortex. A second small cluster was observed in the dorsal cingulate gyrus (Talairach co-ordinate: 9, 23, 20). The final cluster of activity was seen in left post-central gyrus (‘PoCG’, Talairach co-ordinate: −36, −19, −35; Fig. 3) and extended superior and anterior into the superior parietal cortex (Figs 3, 4 and 6). There were no significant areas of activation for the reverse contrast of dehydration >water.

#### Cerebral blood flow

A voxel wise paired contrast of the perfusion values, obtained via the ASL scans, did not reveal any significant differences in resting perfusion for either the contrast of dehydration vs. water, or water vs. dehydration (all *p’s* > *0,05*, uncorrected for multiple comparisons).

#### Association between differences in brain activity, HRV and mood

Peaks of activity occurred on the ventral cingulate gyrus (vCG), the border of the medial orbitofrontal cortex (mOFC) and in the left post-central gyrus (PoCG). To investigate the possible mechanisms behind the observed differences in brain (see above), the difference in brain activity between when participants had, or had not consumed water, was correlated (Pearson’s r) with difference scores (no water minus water) for heart rate variability and mood. Results are displayed in Table 3. Heart rate variability correlated significantly with activity in the mOFC (*r* = 0.78, p < 0.005) and vCG areas (r = 0.77, p < 0.05; Fig. 4); in both instances a smaller increase in R-R interval (i.e a higher heart rate) was associated with lower activity brain activity when no water had been drunk. Similar effects were found for the HRV indices; both the SDNN and RMSSD correlated significantly with activity in the mOFC (*r* = 0.42, p < 0.05 for SDNN and *r* = 0.48, p < 0.05 for RMSSD) and vCG (*r* = 58, p < 0.05 for mOFC and *r* = 0.52, p < 0.05 for vCG) regions, indicating that the hypo-hydration-associated decline in activity may reflect changes in cardiac vagal activity. Overall these findings suggested that the decline in brain activation that was observed when participants didn’t drink reflected autonomic responses to the low-level physiological challenge of hypo-hydration.

**Table 3:** Correlations between differences in brain activation and differences in heart rate variability.

	mOFC	vCG	PoCG
∆ RR interval	0.787**	0.776**	0.145
∆ SD of R-R interval	0.428*	0.580**	0.027
∆ RMSSD	0.486*	0.529*	0.350

Data are Pearson’s correlation coefficient (r) ∆ Difference, **p < 0.005, *p < 0.05, RR, interbeat interval, SDNN, standard deviation of normal to normal R-R interval, RMSSD, root mean square of the standard deviation, mOFC, medial orbitofrontal cortex, PoCG, post-central gyrus, vCG, ventral cingulate gyrus.

To determine whether the observed changes in autonomic and brain functioning have consequences for affect these differences were correlated (Pearson’s r) with ratings of perceived difficulty and mood. (Table 4). The average length of the R-R interval correlated with both anxiety (*r* = −0.61, p < 0.03) and perceived difficulty (*r* = -0.45, p = 0.06); a lower RR interval (i.e higher heart rate) in the no water condition was related to greater anxiety and more perceived effort (Fig. 4). Similar associations were found between the subjective ratings and activity in the mOFC (r = −0.72, p < 0.01 for anxiety; r = −0.60, p < 0.02 for difficulty) and between the subjective ratings and activity in the vCG (r = −0.77, p < 0.007 for anxiety; r = −0.75, p < 0.004 for difficulty); in each case lower activity was associated with more anxiety and greater difficulty. Outside the scanner, composure again correlated with the average length of the RR interval (r = 0.47, p < 0.05), SD of the RR interval (r = 0.44, p < 0.05), and a depressed mood correlated with activity in the vCG (r = 0.75, p < 0.01).

**Table 4:** Correlations between differences in brain activation, hydration parameters and performance and difficulty ratings in the scanner.

	Perceived difficulty	Happy	Energetic	Anxious	Depressed (outside of the scanner)	Energetic (outside of the scanner)	Composed (outside of the scanner)
∆ RR interval	−0.456*	0.086	0.145	−0.613*	0.336	−0.179	0.475*
∆ SD of R-R interval	−0.540*	−0.068	−0.133	−0.746*	0.650*	−0.041	0.445*
∆ RMSSD	−0.298	0.058	−0.195	−0.259	0.382	0.433	−0.129
mOFC	−0.601*	0.278	0.247	−0.722*	0.393	−0.101	0.158
vCG	−0.754*	−0.228	−0.296	−0.774*	0.752*	−0.032	0.114
PoCG	−0.026	−0.036	−0.034	−0.319	0.007	0.471	0.103

*p < 0.05 RR, interbeat interval, SDNN, standard deviation of normal to normal R-R interval, RMSSD, root mean square of the standard deviation, mOFC, medial orbitofrontal cortex, PoCG, post-central gyrus, vCG, ventral cingulate gyrus.

## Results: Study 2

Given the small samples size invariably associated with studies involving neuroimaging a second behavioral study was conducted to replicate and extend the main findings from study 1. Specifically it was hypothesized that declines in HRV would mediate the effect of hypo-hydration on mood.

### Verification of the hydration paradigm

The effect of drinking on percentage total weight loss, perspiration rate and changes in osmolality and thirst were compared across the two conditions using ANOVA. As observed in study 1, participants who did not drink water lost more weight both before (F(1,54) = 15.963, p < 0.0001, η_p_^2^ = 0.227) and after (F(1,54) = 4.705, p < 0.03, η_p_^2^ = 0.087) urination; losing an average of 0.55% of their body mass. In addition, those who didn’t drink had a significant increase in urine osmolality (F(1,54) = 6.994, p < 0.011, η_p_^2^ = 0.117), although the effect of thirst was not significant (F(1,54) = 1.885, p < 0.175, η_p_^2^ = 0.034). Thus in both studies there was evidence that the current paradigm is a valid way of manipulating small changes in hydration without exposing participants to physical activity (Table 1).

### Heart rate variability

The effect of drinking water on heart rate variability was considered by comparing change scores (end of the morning minus baseline) using ANOVA (Table 1; Fig. 2), the findings in study 2 were similar those in study 1. If water was consumed participants had an increase in their average R-R interval (F(1,54) = 13.023, p < 0.001, η_p_^2^ = 0.194), a larger standard deviation of the R-R interval (F(1,54) = 5.187, p < 0.027, η_p_^2^ = 0.088), and a larger root mean squared of standard deviation of R-R interval (F(1,54) = 4.873, p < 0.031, η_p_^2^ = 0.083). Therefore, the studies provided consistent evidence that minor hypo-hydration results in an increased heart rate and decreased heart rate variability.

### Subjective ratings

Data were analysed using ANOVA where change scores were compared across conditions. When anxiety was considered, participants who did not drink water became significantly more anxious (F(1,54) = 4.706, p < 0.034, η_p_^2^ = 0.080). However, the effect was not significant when ratings of depression (F(1,54) = 0.734, p < 0.395, η_p_^2^ = 0.013) and energy (F(1,54) = 0.152, p < 0.698, η_p_^2^ = 0.003) were considered (Table 2).

### Association between HRV and mood

A decrease in average R-R interval was associated with an increase in rating of depression (r = 0.276, p < 0.040), although no association was observed with ratings of anxiety (r = 0.141, p < 0.301) or energy levels (r = 0.008, p < 0.955). A decrease in SDRR was associated with an increase in rating of depression (r = 0.315, p < 0.018) and an increase in anxiety levels (r = 0.335, p < 0.012), but again no association with observed with reported energy (r = 0.104, p < 0.445). RMSSD was not associated with any of the mood measures (anxiety; r = 0.095, p < 0.488, depression; r = 0.215, p < 0.111, energy levels; r = −0.195, p < 0.151).

**Figure 5:**
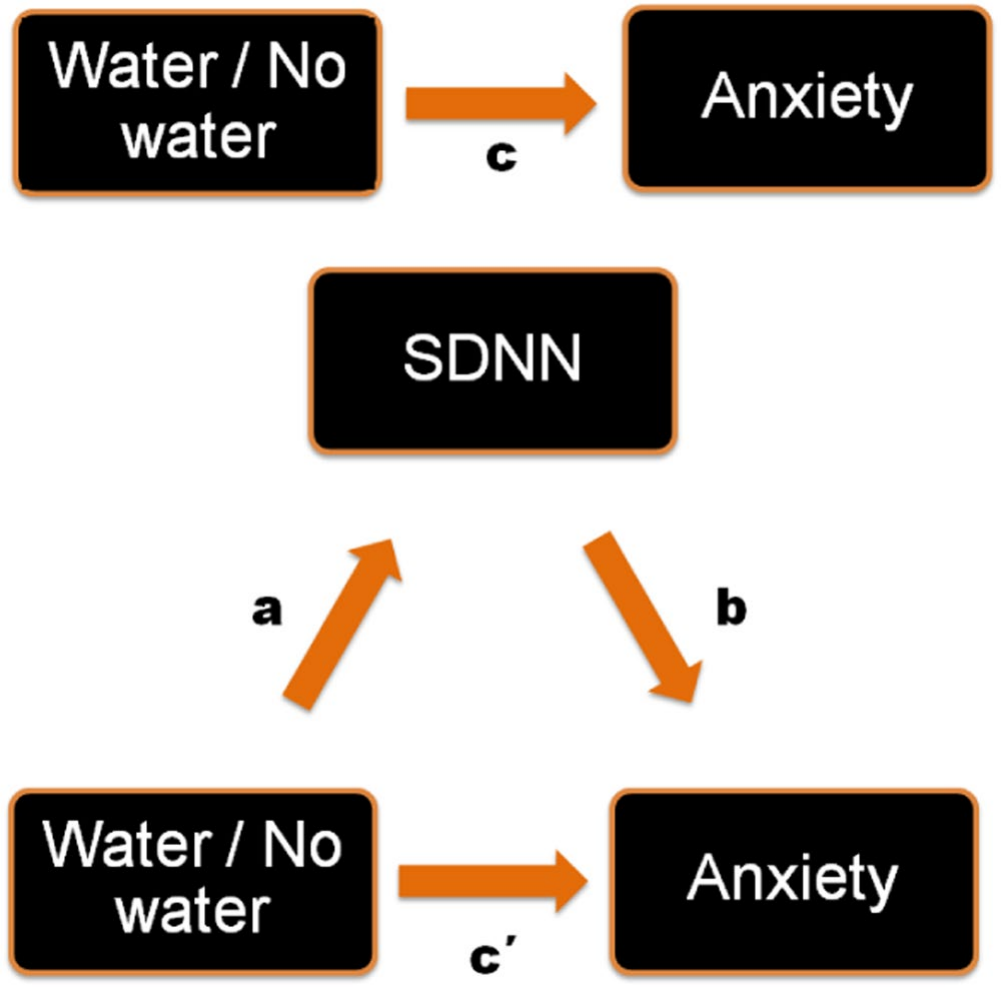
Schematic illustration of the mediation analysis used in study 2. The total effect of water on rating of anxiety was significant (B = 16.321, LLCI 3.729, ULCI 28.913). The direct effect (when the influence of SDNN was considered) was not significant (B = 12.763, LLCI -0.257, ULCI 25.784). When water was not consumed participants were more anxious (F(1,54) = 4.706, p < 0.034). A decrease in HRV (SDRR) was associated with an increase in anxiety levels (r = 0.335, p < 0.012). The indirect effect Water → SDNN → Anxiety was significant (B = 3.557, LLCI 0.212, ULCI 8.321).

To confirm whether changes in HRV mediate the effect of hypo-hydration on mood a mediator analysis was conducted using Hayes PROCESS model 4 (Fig. 5). As anxiety was the only mood measure to show a significant main effect of drinking water this was the dependent variable (Y). Water consumption was the dichotomous independent variable (X), and SDNN was the mediator (M). As reported previously, the total effect of water on rating of anxiety was significant (B = 16.321, LLCI 3.729, ULCI 28.913), however, the direct effect (when the influence of SDNN was considered) was not significant (B = 12.763, LLCI -0.257, ULCI 25.784). The indirect effect Water → SDNN → Anxiety was significant (B = 3.557, LLCI 0.212, ULCI 8.321). This finding suggested that hypo-hydration associated increases in anxiety levels are fully mediated by autonomic adaptations as indexed by a reduction in HRV.

## Discussion

In two studies it is reported that hypo-hydration adversely influenced mood and brain functioning. In study 1, as a result of not drinking water during the experimental protocol, task related activity in the autonomic network of the brain was reduced; specifically activity in the orbito-frontal cortex, ventral cingulate gyrus, dorsal cingulate cortex, hypothalamus, amygdala, right striatum, post-central gyrus and superior parietal cortex was affected. When participants were hypo-hydrated they had a higher HR and lower HRV; effects that correlated with peak activation in the vCG and mOFC. Importantly, these effects were associated with a decline in mood. The deleterious effect of hypo-hydration on HRV was replicated in study 2 where this variable was found to mediate the influence of not drinking water on mood. These observations are consistent with the hypothesis that hypo-hydration elicits a change in autonomic regulatory activity and brain function, with potential adverse consequences for aspects of mood.

In the context of exercise severe hypo-hydration (>2% loss of body weight) elevates HR^1^ but there has been little study of the effect of everyday fluctuations in hydration status on cardiac functioning. The present studies found that in participants at rest, hypo-hydration resulted in a shorter average R-R interval i.e a higher HR (on average 5.8 beats per minute higher) and lower HRV (Fig. 2). Interestingly, limited evidence suggests that drinking water, even in the absence of hypo-hydration, may modulate the cardiac vagal response; 20 and 25 min after drinking 500 ml of water heart rate fell from 67 to 60 bpm and RMSSD increased by 13 ms^15^. This latter finding is important because it might help explain how acute water supplementation can benefit mood, irrespective of hydration status^30^.

**Figure 6:**
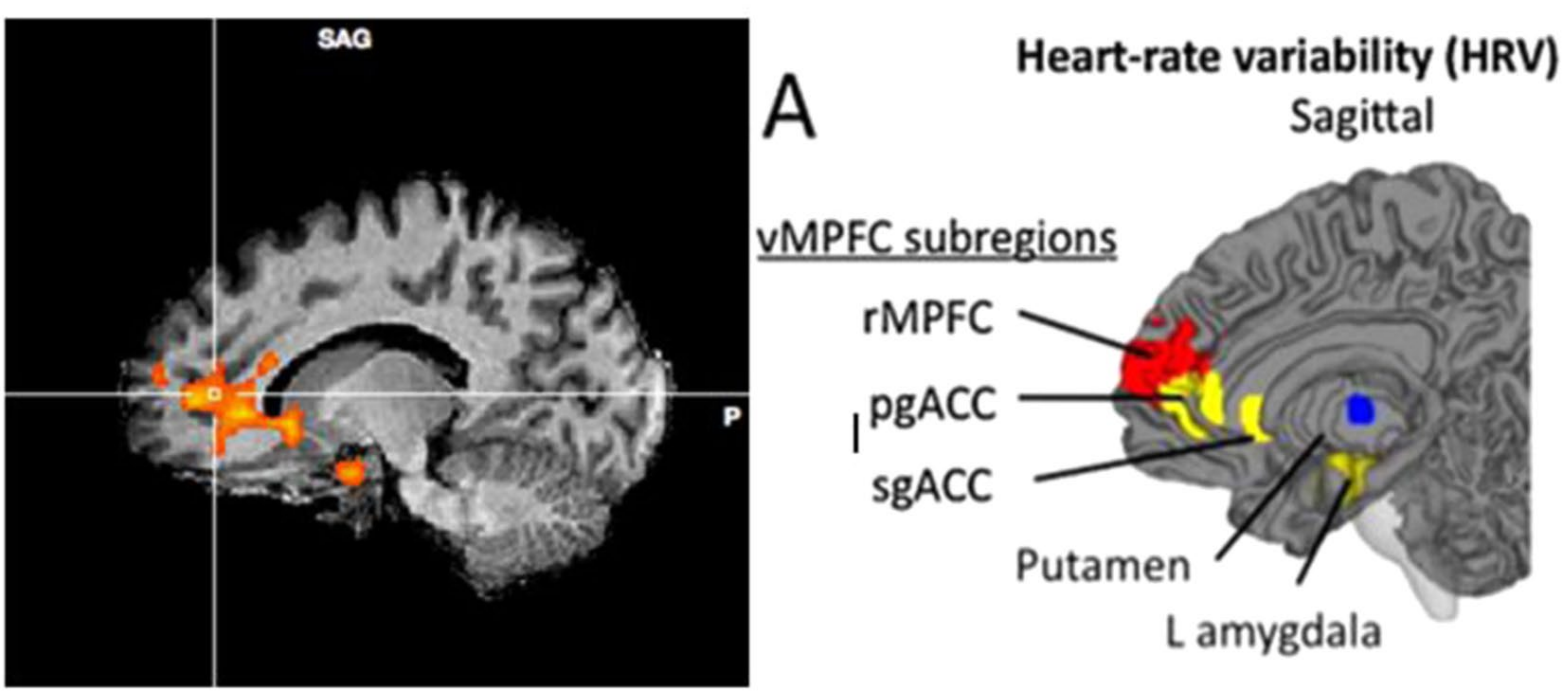
Map showing the brain areas associated with autonomic modulation. Left data from present study. Right areas controlling HRV reproduced from Thayer *et al*. (2012) with permission.

Areas of the brain mediating sympathetic and parasympathetic control of the ANS include regions of the limbic forebrain such as the subgenual and pregenual cingulate, amygdala/ventral striatum and medial prefrontal cortex^20^: as can be seen in Fig. 6, changes in many of the same regions were associated with hypo-hydration. Mathews *et al*.^31^ studied the association between activity in the vCG and peak high frequency power (a HRV index of vagal activity) during a Stroop task; they found a positive association such that greater activity in the vCG was associated with a higher vagal tone. Similarly, in the present study, having higher activity in the vCG and mOFC was associated with greater parasympathetic activity (indexed by the average RR interval, SDRR and RMSSD). These findings indicated that the hydration related differences in brain activity in these regions may be the result of hydration induced autonomic modulation. Interestingly, drinking water both ameliorated the task related decline in brain activity, as indicated by fMRI, and increased HRV. A novel hypothesis is that hypo-hydration induces increased cardiovascular strain, which in turn necessitates a decline in activity in the autonomic network of the brain, effects that could compromise cognitive and affective processing.

Even in a healthy population lower HRV is associated with poorer mood and cognition and higher perceived stress^22^. Similarly, study 1 found that a higher heart rate and lower HRV were associated with increases in anxiety and perceived effort during scanning; these effects were also associated with having a greater deactivation in the mOFC and vCG. In addition, the association between lower HRV and increased anxiety was replicated in study 2. Hypo-activation of the anterior cingulate and frontal cortices is common in a range of anxiety disorders^32^ and during induced anxiety^33^. In addition, interventions known to reduce anxiety increase activity in these regions^34^; it is therefore plausible that the benefits of drinking water reflect similar mechanisms. These observations are important as they may have clinical implications for populations characterized by raised anxiety levels.

Notably, in study 1, mood monitored while participants were in the scanner did not differ according to whether or not they consumed water (Table 2), however, before they left the 30 °C environment (see methods section) there were between condition differences in anxiety; an effect subsequently replicated in study 2. Ambient temperatures in excess of 27 °C exacerbate the effects of dehydration, induce peripheral vasodilatation and result in relative hypovolemia, increasing cardiac strain^1^. Thus, autonomic adaptations that mediate the effects of hydration are likely to be enhanced by a warm environment. It is plausible that by the time participants entered the scanner (on average 22 °C) the exacerbating effect of heat would have been diminished. Supporting this view, Farrell *et al*.^35^ recently reported that regions of the central autonomic network were activated in response to both thermogenic and psychogenic sweating, in particular the anterior cingulate cortex, lentiform nuclei and parietal cortex. In the present study perspiration rate correlated significantly with activity in all three regions of difference; the mOFC, vCG and PoCG (Table S1). If such regions are also involved in hypo-hydration associated cognitive and affective deficits, this might help to explain the effects of the interaction between thermoregulation and hypo-hydration on cognition and mood. Future research might consider replicating these findings using a water deprivation paradigm that doesn’t involve exposure to increased ambient temperatures.

The present findings support the hypothesis that even a relatively minor decline in hydration necessitates a counter-regulatory autonomic response which may have affective consequences. However, an alternative hypothesis might be that, rather than altering neural activity, hypo-hydration reduces cerebrovascular coupling. Given that brain functional imaging methods, such as fMRI, are sensitive to changes in cerebral blood flow (CBF) it is plausible that the observed differences in regional BOLD activity might reflect differences in CBF. Hypo-hydration of 1.3% reduced mean middle cerebral artery blood flow velocity during a cold pressor test, suggesting that the cerebrovascular response to acute stressful stimuli may be altered by hydration status^36^. However, the present study found no effects of hydration on global or regional blood flow using resting state ASL; nonetheless, it remains possible that dynamic cerebrovascular reactivity, rather than resting state auto-regulation, may have been compromised under the present conditions. This remains an important consideration for future research that examines the effects of hydration on brain functioning using imaging.

The limitations of the present study should be considered. Firstly, it is unclear whether hypohydration related changes in anxiety and perceived effort are driving the associated differences in HRV and brain activity, or vice versa. Further research might consider the potential influence of individual differences in afferent baroreceptor activity and interoceptive abilities which has been shown to modulate the processing of emotional information^37,38^, and may be increased following water consumption. A factor that could limit the generalizability of the results is the small sample sizes. Although we were able to replicate findings, the possibility exists that the studies were underpowered to detect some smaller differences in mood (e.g. energy levels/depression). In addition, the sample comprised only young university students and future research may consider different populations who may be more susceptible to the effects of hypohydration; for example children and older adults. Finally, although the majority of studies of HRV have not measured respiration it is potentially a confounding variable. Although there is no reason to believe that respiration varied systematically, such that it would have biased the present findings, it is a question to be addressed in future studies.

In conclusion, the present study reports that when participants consumed water, compared with when they were mildly hypohydrated (0.6% loss in body weight) they had improved mood as indicated by VAS ratings, and increased neural activity as indicated by fMRI when faced with a demanding task. For the first time we have highlighted important regulatory neural mechanisms that may account for the psychological benefits of maintaining hydration status. When water was not consumed more weight was lost, osmolality increased and HRV decreased; effects that predicted a larger task related deactivation in the autonomic network of the brain. Only recently have the interactions between bodily reactions and affective processes begun to be elucidated and such phenomena are still often treated as confounding factors. Indices of ANS function have been used as objective measures of affective states and these responses have been treated as epiphenomena, rather than intrinsic to the cognitive or emotional process. As evidenced here, it is possible that changes in ANS activity have consequences for mood. As such hypo-hydration to the extent that it influences ANS activity per se may have negative consequences for brain functioning and mood.

Given the prevalence of voluntary hypo-hydration these findings have important implications for vulnerable cross-sections of the population. Furthermore, the psychological benefits of drinking may have important implications for populations, such as older adults and children^39^ who are at a significant risk of dehydration. The fact that mild changes in hydration affected cardiovascular functioning is also a concern; the HR of participants was on average 5.8 beats per minute lower when they had consumed water. Given that there is a progressive increase in the risk of heart-disease as resting heart rate increases^40^, and hyper-osmolarity predicted a 4.3-fold increased risk of cardiovascular mortality^41^, maintaining adequate hydration may be an important factor for maintenance of cardiovascular health. In general, repeated low level physiological challenges such as hypo-hydration may overtime increase allostatic load, predisposing to possible negative health outcomes.

## Methods

### Sample characteristics (Study 1)

The sample size was based on the expected power for a hypothesized within participants effect. Total sample size was calculated using G*power based on the following parameters: one group, two within subject levels (Water/No water) with an expected correlation of 0.6, α = 0.05, a two-tailed test, and 80% power to detect a medium to large sized effect (Cohen’s f2 = 0.4). This gave an estimated N of 12 which is in line with previous studies that have examined the neural effects of hydration e.g.^26^.

Twelve healthy young males (average age 23.3 years (19–34), average BMI 25.4 (19–34)), gave their written informed consent after being screened for MRI safety. Participants were excluded if they had any health complaint that would affect cardiovascular functioning such as diabetes or hypertension. Similarly, anyone with a neuropsychological illness was also excluded as were those taking medication. All participants were right handed, non-smokers; before the start of the study they were asked to refrain from drinking alcohol for at least 24 hours and told to fast and avoid any beverages for at least twelve hours.

### Sample characteristics (Study 2)

The sample size was based on the expected power for a hypothesized between participants effect. Total sample size was calculated using G*power based on the following parameters that were deduced from study 1: two groups (Water/No water), α = 0.05, a two-tailed test, and 80% power to detect a medium to large sized effect (Cohen’s f2 = 0.4). This gave an estimated N of 52.

Twenty-five males and thirty-one females (average age 21.0 years (18–28), average BMI 23.3 (17–35)), gave their written informed consent and were randomly allocated to either the water or no water condition (Water 15 F, 13 M, No water 16 F, 12 M). As it has been suggested that gender may be a determining factor in the correlation between hypo-hydration and its psychological consequences^2^, this factor was initially considered. Preliminary analysis found no significant interactions between gender and water consumption for any of the dependent variables (all p > 0.1). Exclusion criteria and pre-study instructions were the same as for study 1.

### Procedure (Study 1)

On two occasions participants attended the laboratory that was heated to 30 degrees. Using a repeated measures design, in a randomly derived order, they received either two 150 ml glasses of water or nothing (Fig. 1). Upon arriving at the laboratory, participants were asked to provide a urine sample and were asked to completely empty their bladder, following which they were weighed and their body temperature measured. They were then fitted with a RS800 Polar heart rate monitor electrode transmitter belt (T61) and a Polar RS800 HR monitor (Polar Electro, Kempele, Finland) that was used to collect interbeat interval measurements at a sampling rate of 1000 Hz, while participants rested in a seated position. This instrument has been previously validated for the accurate measurement of R-R intervals and for analysing Heart Rate Variability (HRV)^42^. Participants were then provided with a standard breakfast consisting of 50 g of Quaker Oat So Simple Original Porridge (187 kcal, 2.9 g fat, 10.6 g sugar, 0.11 g salt) plus 150 ml of either decaffeinated tea or coffee. After breakfast participants were weighted again, and rated their mood and thirst. After this they were allowed to rest while either watching TV or reading. Three hours later participants were asked to provide another urine sample and again had their HRV, body temperature and weight measured, and rated their mood and thirst. Finally, they were escorted to the MRI laboratory for the scanning procedure. The procedure was approved by Swansea University ethics committee (ref: 01.01.2015.1) and carried out in accordance with the principles laid down by the declaration of Helsinki 2013. ClinicalTrials.gov Identifier: NCT03525470 (15/05/18).

### Procedure (Study 2)

The procedure for study 2 was identical with the exception that it employed a between participants rather than within subject design. In addition, participants did not complete the fMRI protocol. The procedure was approved by Swansea University ethics committee (ref: 01.03.2018.1) and carried out in accordance with the principles laid down by the declaration of Helsinki 2013. These data were collected as part of a larger trial: ClinicalTrials.gov Identifier: NCT02671149 (02/02/16).

### Mood (Study 1 and Study 2)

Mood measures were identical across both studies. Participants were asked to report on visual analogue scales how they felt “at this moment” using visual analogue scales with pairs of adjectives at the ends of 100 mm lines; Composed/Anxious; Elated/Depressed; Energetic/Tired; as described by McNair and Lorr^43^.

### Thirst (Study 1 and Study 2)

In both studies, participants were asked to responds to the question “how thirsty are you feeling right now” on a single 100 mm visual analogue scale anchored by “Not at all” and “Extremely”.

### Osmolality (Study 1 and Study 2)

The osmolality of urine was assessed using an Osmomat 3000 freezing point osmometer (Gonotec GmbH, Berlin, Germany).

### Body temperature (Study 1)

Body temperature was measured using a TH8 Infrared Ear Thermometer (Radiant Innovation, Taiwan).

### Body mass (Study 1 and Study 2)

Body mass was measured using an electronic scale (Kern KMS-TM, Kenr and Sohn GmbH, Germany) that, to avoid problems associated with movement, took 50 assessments over a 5 second period and produced an average value. It was sensitive enough to weigh to within 5 grams (17% of an ounce) and could pick up over short periods changes in body mass due to breathing and perspiration. Participants were weighed on arrival, both before and after breakfast, and again at the end, both before and after urination.

### Scanning procedure (Study 1)

**Figure 7:**
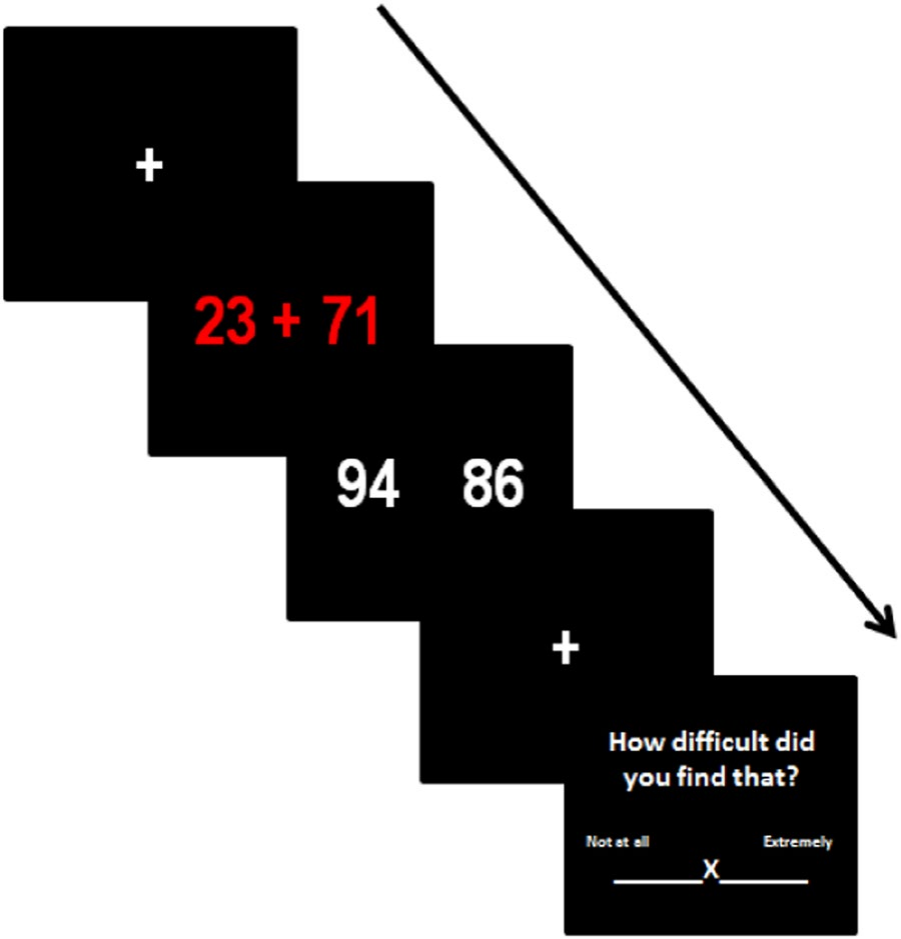
Arithmetic task and Visual analogue scale example stimuli. The crosses are fixation points when otherwise the screen is blank. At the third image the participant indicated whether the right or left number was correct by pushing a button.

Participants were presented with an arithmetic task, modified for use in the scanner, similar to the Paced Auditory Serial Addition Task: a ‘stressful’ task that measures calculation ability and is known to elicit an autonomic response^44^. Pairs of two digit numbers appeared on a screen in red and participants were required to mentally add or subtract the numbers. After 2 seconds the screens was removed and a second screen appeared containing a correct and an incorrect answer. Participants were required to press either a left or right button to indicate which answer was correct (Fig. 7). The speed of presentation is designed to be just at the level that it is possible to perform the task although it required mental effort and was ‘stressful’. This allowed investigation of areas of the brain associated with working memory but also those associated with emotional arousal. Data analysed from the task were number of missed responses, the number of correct responses and average reaction time in milliseconds (on the trials that participants responded). Three blocks of four minutes were performed. At the end of each block four visual analogue scales appeared and participants were asked to rate, using the left and right key to move a cursor, how difficult they found the proceeding block and how happy, energetic and anxious they felt at that moment. The visual analogue scales were rated on a scale of 1–100.
